# A Novel Null Allele of the RAPH Blood Group System

**DOI:** 10.1111/trf.18304

**Published:** 2025-06-13

**Authors:** Lea Wörner, Gabi Rink, Annette Stürtzel, Sina Rothenberger‐Mürb, Susanne Seyboth, Judith Küthe‐Rieger, Albrecht Leo, Peter Bugert

**Affiliations:** ^1^ Institute of Transfusion Medicine and Immunology Heidelberg University, Medical Faculty Mannheim, German Red Cross Blood Service Baden‐Württemberg–Hessen Mannheim Germany; ^2^ German Red Cross Blood Service Baden‐Württemberg–Hessen Institute of Transfusion Medicine and Immunohematology Baden‐Baden Germany; ^3^ Institute for Clinical Transfusion Medicine and Cell Therapy Heidelberg Germany

**Keywords:** blood group panel sequencing, CD151, MER2, RAPH (ISBT025) blood group system

## 1 BRIEF BACKGROUND

The multipass membrane glycoprotein CD151 is the molecular basis of the RAPH (ISBT 025) blood group system with the high prevalence antigen MER2.^1^ The ISBT allele table (Version 5.0, 30‐OCT‐2020) includes three missense single nucleotide variant (SNV) alleles, each leading to an amino acid exchange at positions 165, 171, and 178, and causing the MER2− phenotype.^2^ The RAPH*01 N.01 null allele with a frame‐shift mutation (c.383_384insG) leads to a CD151 protein truncated at amino acid position 128 and the RAPH_null_ phenotype.

Here, a blood sample of a 17‐year‐old male of Turk ethnicity with the underlying disorders of unilateral renal agenesis, hypertension, and chronic kidney disease was sent to our laboratory for compatibility testing. The sample was typed as O RhD‐positive (O CcD.ee). Antibody screen and identification revealed weakly positive reactions in most test cells with negative auto control and negative direct antiglobulin test, suggestive of an irregular antibody directed against a high prevalence blood group antigen. In our reference laboratory, a wide variety of test cells negative for high prevalence blood group antigens and neutralization with soluble recombinant blood group antigens (rBGA) could not elucidate the specificity of the antibody. The corresponding antigen did not seem to be enzyme‐sensitive (Papain). In addition, none of the blood group gene variants underlying lack of high prevalence antigens of various blood group systems (DO, YT, DI, KN, KEL, LU, VEL, CH/RG) typed by in‐house PCR methods were identified. Therefore, all known blood group genes (ISBT 001 to 047) were analyzed in the patient by the use of next generation sequencing (NGS).

## 2 BRIEF METHODS

Genomic DNA was sequenced according to NGS standard protocols for amplicon‐based library generation with the iSeq 100 system (Illumina Inc., Berlin, Germany). Panel sequencing included all exons of the blood group genes encoding the systems ISBT 001 to 047. For data analysis, the Variant Interpreter (Illumina) and the Integrative Genomics Viewer (IGV) tools were used. Genotyping of the *CD151* c.346C>T variant was performed according to standard PCR‐SSP protocol with forward primers for the wild type allele (5’‐CTCGCCTACGCCTACTACC‐3′) and the variant allele (5’‐CTCGCCTACGCCTACTACT‐3′), a reverse primer (5’‐CCAGTGGCACATTGGTTAGC‐3′), and primers for an internal control amplified from the *HBB* gene.^3^

## 3 RESULTS

**FIGURE 1:**
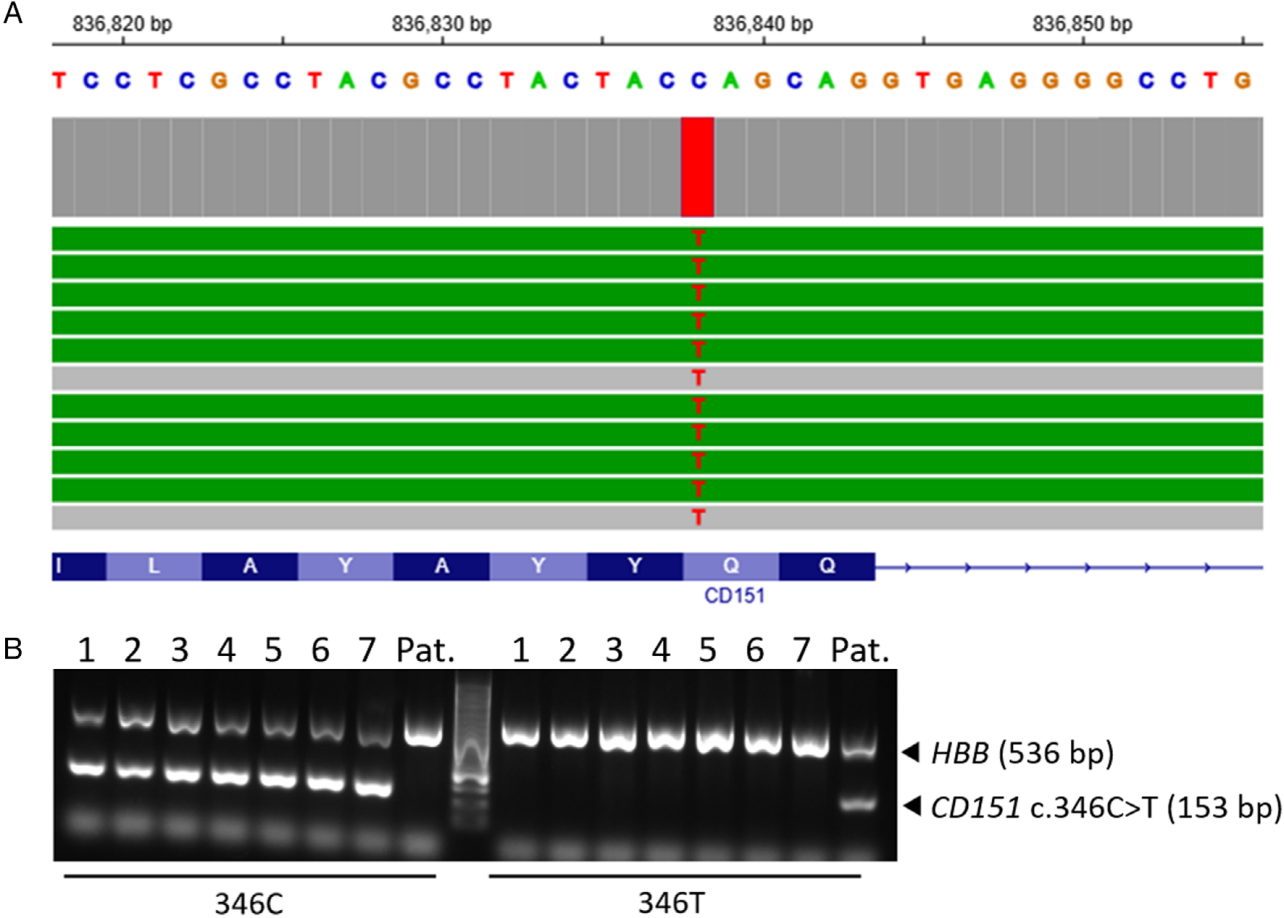
Identification of the novel *CD151* c.346C>T mutation in the patient (Pat.). (A) Panel sequencing showed a homozygous SNV in *CD151* exon 5 at position c.346 (hg38 chromosome 11: G.836,838) with T in 263 of 265 reads (99.2%). The C>T change results in a premature stop codon (CAG>TAG; p.Gln116Ter). (B) PCR‐SSP confirmed the patient's genotype *CD151* c.346 T and unrelated blood donors (1–7) were negative for the T variant.

Panel sequencing of the genes encoding the blood group systems 001 to 047 revealed a homozygous nonsense variant (c.346C>T; p.Gln116Ter) in exon 5 of *CD151* (Figure 1A). The patient's genotype for the variant was confirmed by PCR‐SSP including unrelated blood donors as negative controls (Figure 1B). The premature stop codon caused a truncated CD151 protein, presumably negative for the high prevalence antigen MER2. No further variant detected in panel sequencing gave a hint for a negative high prevalence antigen.

## 4 BRIEF SUMMARY

An allo‐antibody to a high prevalence antigen was found in a patient with chronic kidney disease. Panel sequencing of the blood group genes identified the novel *CD151* c.346C>T nonsense SNV (p.Gln116Ter) as the most probable explanation for the lack of a high prevalence antigen. The variant is not listed in the databases (dbSNP, gnomAD).^4^, ^5^ The CD151 protein (253 amino acids) is truncated at amino acid 116 and all amino acids (position 165, 171 and 178) defining the MER2 antigen are missing. Thus, homozygosity of the c.346 T allele (RAPH*01 N.02) in the patient strongly suggested the RAPH_null_ (MER2–) phenotype with an allo‐antibody against the MER2 antigen. Because the patient's blood sample was limited we could send only a small sample to the International Blood Group Reference Laboratory (IBGRL; Bristol, UK). The patient's cells were confirmed to be MER2‐. However, there was only sufficient sample to type the patient's cells with one example of anti‐MER2 to determine this phenotype. A confirmation of the allo‐antibody specificity was also hampered by the limited patient's plasma. As reported before, the RAPH_null_ phenotype is associated with renal failure^1^ and the patient's clinical diagnosis included unilateral renal agenesis and chronic kidney disease.

## CONFLICT OF INTEREST STATEMENT

The authors have disclosed no conflict of interest.
